# Trends in lung cancer survival: superior outcomes in US Veterans compared with the general population

**DOI:** 10.1093/jncics/pkag054

**Published:** 2026-05-21

**Authors:** Nikki E Rossetti, Brendan T Heiden, Daniel B Eaton, Steven Tohmasi, Nahom Seyoum, Theodore Thomas, Martin Schoen, Whitney S Brandt, Sara Malone, Yan Yan, Ana Baumann, Su-Hsin Chang, Mayank Patel, Daniel Kreisel, Ruben Nava, Bryan Meyers, Benjamin Kozower, Varun Puri

**Affiliations:** Department of Surgery, Washington University School of Medicine, St. Louis, MO, United States; Department of Surgery, Washington University School of Medicine, St. Louis, MO, United States; Department of Surgery, St. Louis VA Medical Center, St. Louis, MO, United States; Department of Surgery, St. Louis VA Medical Center, St. Louis, MO, United States; Department of Surgery, Washington University School of Medicine, St. Louis, MO, United States; Department of Surgery, Washington University School of Medicine, St. Louis, MO, United States; Department of Medicine, Washington University School of Medicine, St. Louis, MO, United States; Department of Medicine, St. Louis VA Medical Center, St. Louis, MO, United States; Department of Medicine, St. Louis VA Medical Center, St. Louis, MO, United States; Department of Medicine, Saint Louis University School of Medicine, St. Louis, MO, United States; Department of Surgery, Washington University School of Medicine, St. Louis, MO, United States; Department of Surgery, Washington University School of Medicine, St. Louis, MO, United States; Department of Surgery, Washington University School of Medicine, St. Louis, MO, United States; Department of Surgery, Washington University School of Medicine, St. Louis, MO, United States; Department of Surgery, Washington University School of Medicine, St. Louis, MO, United States; Department of Surgery, St. Louis VA Medical Center, St. Louis, MO, United States; Department of Surgery, Washington University School of Medicine, St. Louis, MO, United States; Department of Surgery, Washington University School of Medicine, St. Louis, MO, United States; Department of Surgery, Washington University School of Medicine, St. Louis, MO, United States; Department of Surgery, Washington University School of Medicine, St. Louis, MO, United States; Department of Surgery, Washington University School of Medicine, St. Louis, MO, United States

## Abstract

**Background:**

Non-small cell lung cancer (NSCLC) remains the leading cause of cancer-related mortality in the United States, but survival outcomes are shifting with evolving therapies and screening. Recent findings suggest that survival improvements vary across health-care systems, with favorable trends observed in the Veterans Health Administration (VHA). We sought to compare NSCLC survival trends across US health systems.

**Methods:**

We conducted a retrospective cohort study of patients with NSCLC (2007-2019) using data from the VHA and the National Cancer Database (NCDB), representing the non-VHA US general population. The primary exposure was health system (VHA vs non-VHA), and the primary outcome was 3-year all-cause mortality. Multivariable Cox models estimated annual adjusted overall survival (OS), with interaction terms for health system and stage.

**Results:**

Among 1 463 996 patients (VHA n = 79 027; NCDB n = 1 38 4 969), adjusted 3-year OS increased from 24% to 51% (VHA) and from 24% to 41% (non-VHA) between 2007 and 2019. The VHA survival advantage persisted in analyses stratified by stage (all *P*-values <.0001) and when analyses were limited to NCDB patients with Medicare or private insurance (51% vs 42% in 2019; *P* < .0001)

**Conclusions:**

In this nationally representative study, 3-year OS among patients with NSCLC improved between 2007 and 2019, with larger and more rapid gains observed within the VHA compared with non-VHA settings. These findings suggest that lung cancer care delivered within an integrated, publicly funded system is associated with greater and more rapidly improving survival than care delivered across a heterogeneous mix of US health-care delivery settings represented in the NCDB.

## Introduction

Lung cancer remains the leading cause of cancer-related mortality in the United States, with persistently low overall survival rates despite recent therapeutic advances.^1^ Over the past 2 decades, the management of non-small cell lung cancer (NSCLC) has undergone substantial transformation, including advances in early detection via lung cancer screening,^1^^,^^2^ improvements in surgical techniques,^3^ the expanded use of stereotactic body radiotherapy (SBRT) for early-stage disease,^4^ and the introduction of targeted systemic agents and immunotherapies for advanced-stage disease.^5^^,^^6^ These advances have reshaped the clinical landscape, but lung cancer remains a high-burden, high-cost disease with substantial variation in survival across care settings.^1^^,^^2^

Given the complexity of lung cancer care and the rapid pace of innovation, systematic interventions such as care coordination, access to guideline-concordant treatment, and multidisciplinary clinical care have become increasingly important determinants of outcomes.^7-10^ Although individual clinical decisions remain vital, population-level outcomes are shaped by the broader structure in which care is delivered.^7^^,^^11^^,^^12^ Lung cancer may serve as a bellwether for evaluating how different health systems manage rapidly evolving clinical paradigms, highlighting both the opportunities and limitations of various care delivery models.^9^^,^^10^ Understanding the impact of these system-level differences is critical for informing national policies.

The Veterans Health Administrations (VHA) is the largest integrated health-care system in the United States. Several recent studies have demonstrated that the VHA delivers equal or better care than non-VHA systems across several quality domains.^13^ In particular, the adoption of lung cancer screening programs has been far more robust in the VHA. Although the lung cancer screening rates remain below 20% in the US general population,^14^ it is estimated that screening rates are higher among eligible veterans.^15^ A recent study by Moghanaki^16^ of more than 50 000 veterans diagnosed with lung cancer between 2010 and 2017 demonstrated a marked shift toward earlier-stage diagnosis, attributable in part to increased screening rates within the VHA.^15^ Furthermore, in a large national comparative analysis, our group showed that the VHA outperformed all other major US insurance models in early-stage NSCLC diagnosis after the introduction of national screening guidelines,^17^ suggesting that higher screening uptake and integrated care delivery translated into earlier detection at the population level. Despite staggering differences in early-stage diagnoses based on health-care system, to our knowledge, no prior studies have directly compared modern lung cancer survival outcomes for patients treated in the VHA with those of the US general population across all stages of NSCLC.

Building on these findings, the current study aimed to compare NSCLC outcomes among patients treated in the VHA with the US general population. We analyzed 3-year overall survival trends among patients diagnosed with NSCLC between 2007 and 2019, comparing survival outcomes in the VHA with those in the National Cancer Database (NCDB), a nationwide hospital-based registry representing patients in the US general population with diverse insurance types and clinical settings. We hypothesized that survival would improve over time in both populations based on recent advances in lung cancer care across multiple treatment paradigms. Furthermore, we posited that survival gains might be greater in the VHA, potentially reflecting advantages of its integrated infrastructure, including coordinated care delivery, unified clinical information systems, and multidisciplinary oncology care.

## Methods

### Informed patient consent

The research protocol for this study was approved by the St. Louis VHA’s Research and Development Committee (#1214632, August 2, 2019) and Institutional Review Board, which waived the requirement for signed informed consent given the deidentified nature of the analyses. This study was conducted in accordance with the Declaration of Helsinki (revised 2013).

### Study population

This retrospective cohort study used data from the 2021 participant user file of the NCDB (release year 2023) and the VHA Corporate Data Warehouse, as collated in the VA Informatics and Computing Infrastructure (VINCI). The NCDB is a national cancer registry that collects cancer data from more than 1500 hospitals and captures more than 70% of newly diagnosed cancers in the United States. As such, the dataset is generally representative of cancer care in the general US population.^18^ For the VHA analysis, NSCLC cases were identified using International Classification of Diseases (ICD) for Oncology diagnosis codes. Cancer staging data were extracted and categorized according to the American Joint Commission on Cancer (AJCC) Staging Manual, 7th and 8th editions. The study included all patients diagnosed with NSCLC between January 1, 2007, and December 31, 2019. Patients with unknown stage at diagnosis were excluded. The VHA cohort was established on September 30, 2024. The NCDB participant user file was acquired July 7, 2023.

### Covariates

Patient-level covariates included age, sex, race, and NSCLC stage at the time of diagnosis. Charlson-Deyo comorbidity scores were directly obtained from the NCDB participant user file for NCDB patients. For the VHA cohort, Charlson-Deyo scores were calculated based on documented medical comorbidities using ICD-9 and ICD-10 diagnosis codes documented during the 3 years preceding the date of diagnosis. The methods used to curate this VHA cohort have been described and validated previously by our group^19-21^ and were developed by a multidisciplinary research team over several years. The NCDB data elements have also been described previously.^18^

### Outcomes

The primary outcome was 3-year all-cause mortality from the date of NSCLC diagnosis. For the VHA, mortality was determined using the VHA Death Ascertainment File, a validated composite of federal and VA data sources with near-complete concordance with the National Death Index.^22^^,^^23^ Overall survival was assessed at 3 years to ensure equivalent follow-up across all included diagnostic years (ie, as 2019 cases in the NCDB had only 3 years of available follow-up). Patients were censored on the date of last follow-up (December 31, 2022). Survival data were directly abstracted from the NCDB.

### Statistical analysis

Descriptive statistics were calculated for baseline cohort characteristics. Comparisons between groups (VHA vs NCDB) were made using *t* tests for continuous variables and χ^2^ tests for categorical variables. A 2-sided *P* < .05 was considered statistically significant.

Multivariable Cox proportional hazards models were used to estimate annual calendar year-specific 3-year overall survival rates, with administrative censoring on December 31, 2022. Adjusted overall survival estimates were calculated for a standardized reference patient defined as a 65-year-old White male with no medical comorbidities. To account for potential nonlinear trends over time, restricted cubic splines were applied to the time variable with knots at the years 2008, 2013, and 2018, corresponding to the 10th, 50th, and 90th percentiles of the data distribution. A spline × health system interaction term was included to evaluate differential temporal survival trends based on health system.

In a sensitivity analysis designed to reduce heterogeneity related to insurance status and access to care within the US general population, we restricted the NCDB cohort to patients insured by Medicare or private payors, which are considered to be the two most comprehensive insurance models in the general population.

As an additional sensitivity analysis, we incorporated NCDB facility designation into the model, categorizing sites as Community Cancer Programs, Comprehensive Community Cancer Programs, Academic/Research Programs, and Integrated Network Cancer Programs alongside VHA facilities as a fifth category. A spline × facility interaction term was included to assess differences in survival trajectories across facility types.

All analyses were performed using SAS version 9.3 (SAS Institute, Cary, NC).

## Results

### Baseline cohort characteristics

A total of 1 463 996 patients diagnosed with NSCLC between 2007 and 2019 were included (VHA n = 79 027 and NCDB n = 1 384 969 patients). Summary statistics for both cohorts are provided in Table 1. Patients in the VHA cohort were slightly older (mean = 69.4 vs 68.8 years, *P *< .0001), more likely to be male (97.5% vs 52.3%, *P* < .0001), and had a higher comorbidity burden (CCI ≥3: 24.0% vs 5.7%, *P* < .0001). The VHA cohort also included more non-White patients (19.2% vs 15.3%, *P* < .0001). In both cohorts, the majority of patients were diagnosed at stage IV; however, stage I disease was more common in the VHA (29.6% vs 27.9%) and stage IV disease was less common (38.3% vs 41.6%), reflecting a modest shift toward earlier-stage diagnosis in the VHA (*P* < .0001).

**Table 1.** Patient characteristics of the overall unmatched study cohorts.

Variable	**VHA (N = 79** **027)**	**NCDB (N = 1** **384** **969)**	*P*
Age in years, mean (SD)	69.40 (8.95)	68.82 (10.56)	<.0001
Race, n (%)			<.0001
Non-White	15 174 (19.20%)	211 150 (15.25%)	
White	63 853 (80.80%)	1 173 819 (84.75%)	
Sex, n (%)			<.0001
Female	2016 (2.55%)	660 268 (47.67%)	
Male	76 999 (97.45%)	724 701 (52.33%)	
Charleson-Deyo Comorbidity Score, n (%)			<.0001
0	26 037 (32.95%)	793 585 (57.30%)	
1	20 503 (25.94%)	368 817 (26.63%)	
2	13 520 (17.11%)	143 606 (10.37%)	
3+	18 967 (24.00%)	78 961 (5.70%)	
Year of diagnosis, mean (SD)	2012.5 (3.53)	2013.2 (3.68)	<.0001
NSCLC stage, n (%)			<.0001
I	23 375 (29.58)	386 609 (27.91)	
II	7273 (9.20)	125 679 (9.07)	
III	18 140 (22.95)	297 167 (21.46)	
IV	30 239 (38.26)	575 514 (41.55)	

### Primary overall survival analysis

**Figure 1.**
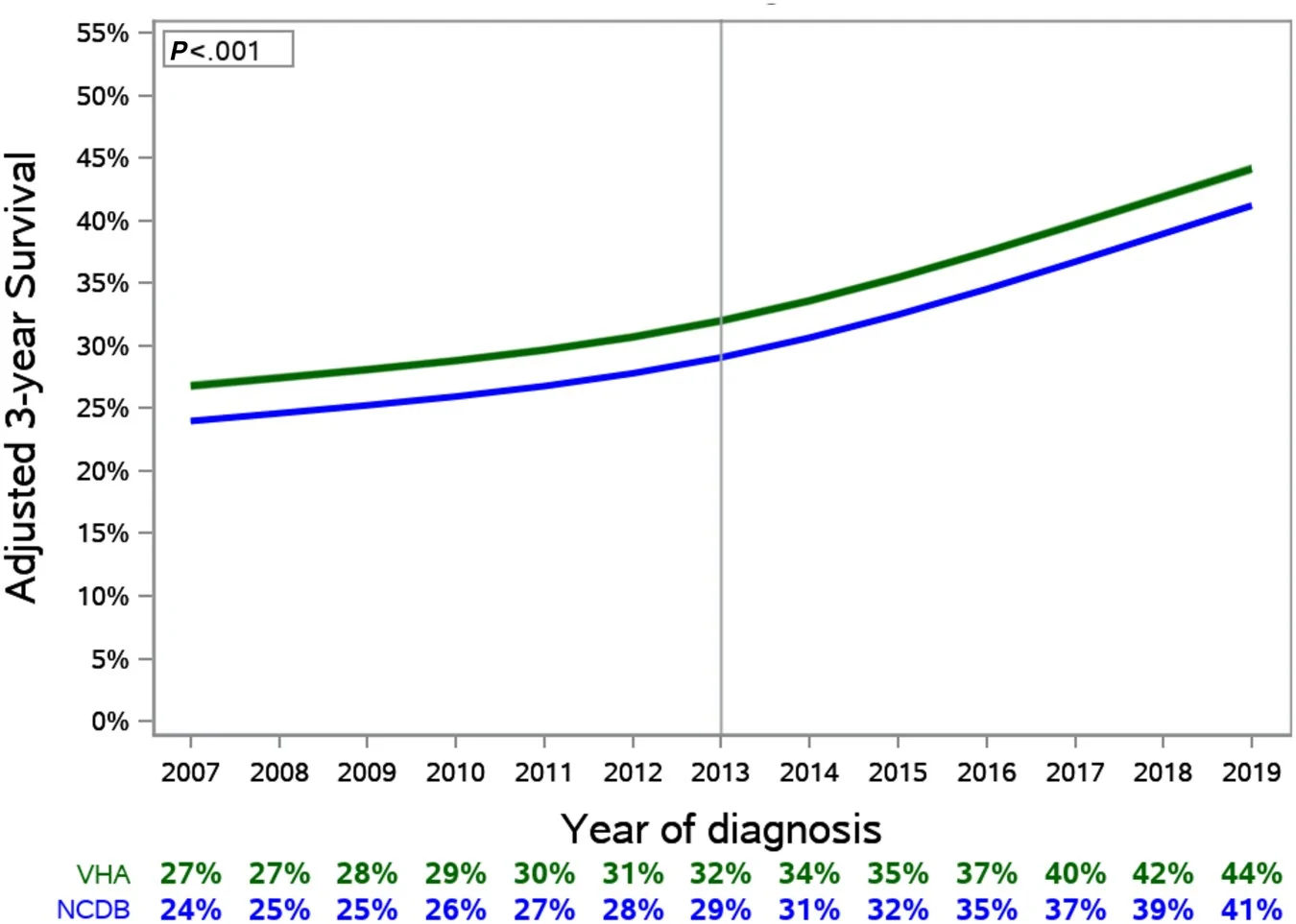
Adjusted 3-year overall survival among patients with non–small cell lung cancer (NSCLC) in the Veterans Health Administration (VHA) and National Cancer Database (NCDB), 2007 to 2019. Estimates are derived from multivariable Cox proportional hazards models for a standardized reference patient (65-year-old White male with no comorbidities) and reported with 95% confidence interval. *P*-value corresponds to the interaction between health system and year of diagnosis (spline × health system), reflecting differences in survival trends over time.

Adjusted 3-year overall survival improved over time for both cohorts (VHA and NCDB) but with significantly greater gains in the VHA population (Figure 1). For patients diagnosed in 2007, survival rates were 24% in both cohorts. However, by 2019, survival reached 51% in the VHA compared with 41% in the NCDB, representing a 10% absolute survival advantage favoring the VHA.

Survival gains began to diverge notably around 2012, with the gap continuing to widen through the end of the study period. The interaction between year of diagnosis and health system (VHA vs NCDB) was highly significant (*P* < .0001), confirming that survival trends over time differed between the two populations.

### Survival trends stratified by NSCLC stage

**Figure 2.**
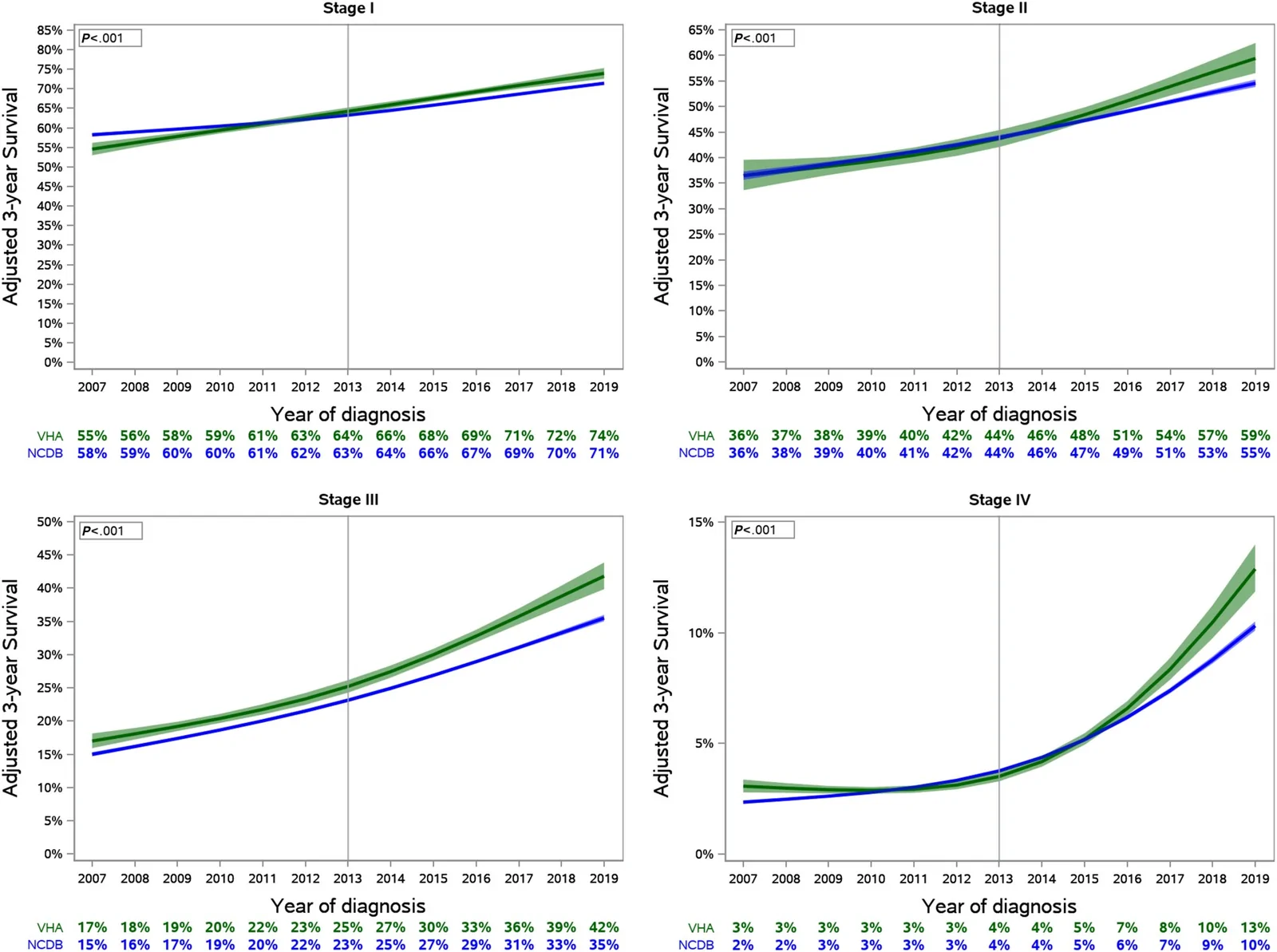
Adjusted 3-year overall survival by stage at diagnosis (Panel A—stage I; Panel B—stage II; Panel C—stage III; and Panel D—stage IV) among patients with NSCLC in the VHA and NCDB, 2007 to 2019. Estimates are reported with 95% confidence intervals. *P*-value corresponds to the interaction between health system and year of diagnosis (spline × health system), reflecting differences in survival trends over time.

To evaluate whether survival trends across groups varied by stage of disease at diagnosis, adjusted 3-year overall survival was analyzed separately for stages I-IV (Figure 2). In each stage-stratified analysis, adjusted survival improved over time in both cohorts, with consistently greater gains observed in the VHA population across all stages. From 2007 to 2019, stage I survival increased from 55% to 74% in the VHA and from 58% to 71% in the NCDB (interaction *P* < .0001); stage II survival increased from 36% to 59% in VHA and from 36% to 55% in the NCDB (interaction *P* < .0001); stage III survival increased from 17% to 42% in the VHA and from 15% to 35% in the NCDB (interaction *P* < .0001); and stage IV survival increased from 3% to 13% in the VHA and from 2% to 10% in the NCDB (interaction *P* < .0001).

### Sensitivity analysis: insurance restriction

**Figure 3.**
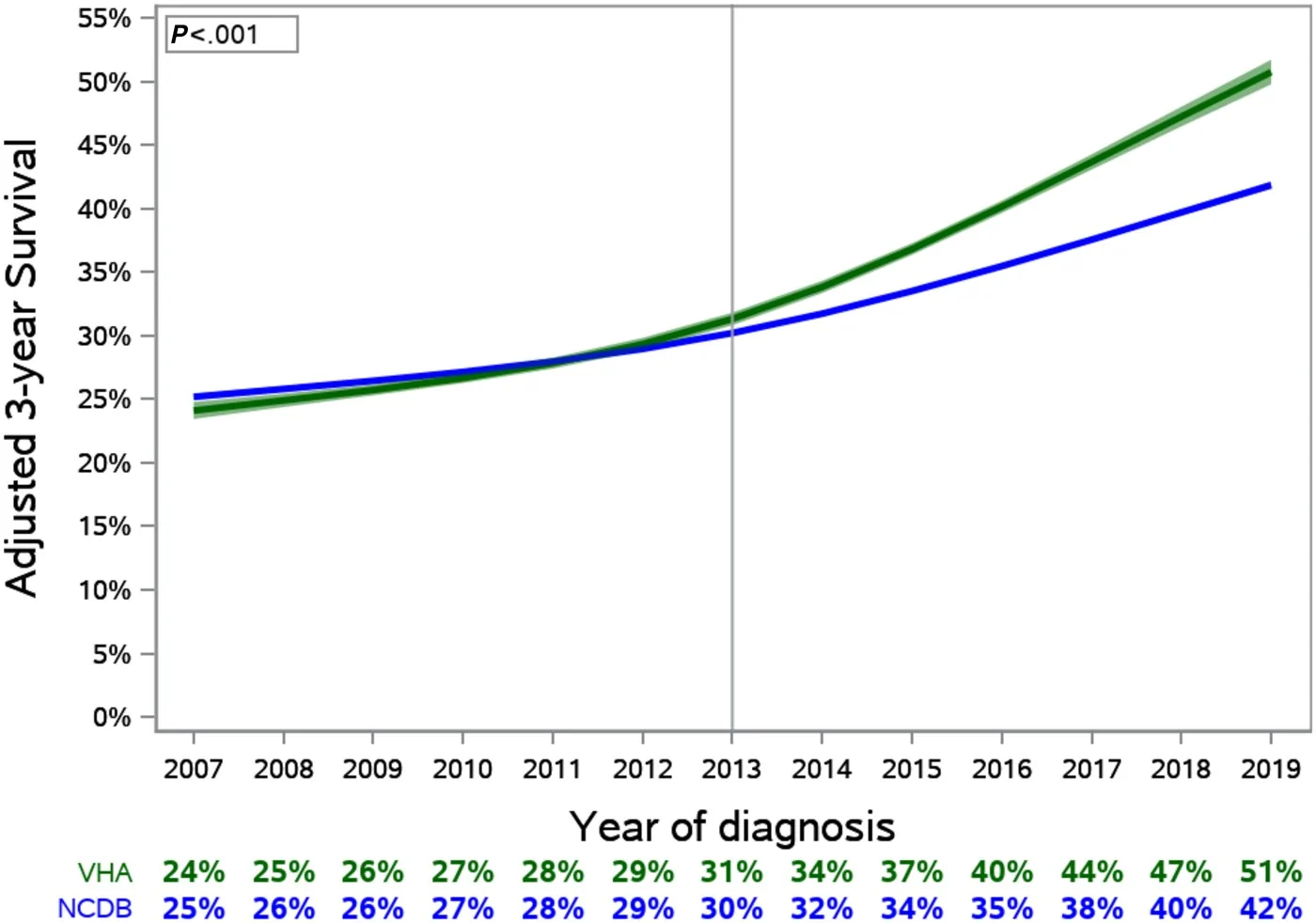
Adjusted 3-year overall survival among patients with NSCLC in the VHA and NCDB restricted to those with Medicare or private insurance, 2007 to 2019. Estimates are reported with 95% confidence intervals. *P*-value corresponds to the interaction between health system and year of diagnosis (spline × health system), reflecting differences in survival trends over time.

In a sensitivity analysis restricting the NCDB cohort to patients insured by Medicare or private payors (Figure 3), survival trends remained consistent with the primary analysis. The year × health system interaction term remained significant (*P* < .0001), and the VHA survival advantage in later years persisted.

### Sensitivity analysis: facility type

In an additional sensitivity analysis incorporating NCDB facility designation (Figure S1), survival improved across all facility types (*P* < .0001), with a significant year × facility interaction (*P* < .0001). Among 1 384 969 total NCDB patients, Academic/Research programs account for 429 374 (31%), whereas the remaining majority (69%) were treated in Comprehensive Community (n = 564 039, 40.7%), Integrated Network (n = 278 715, 20.1%), or Community (n = 106 207, 7.7%) programs. Summary statistics for each facility type are provided in Table S1. By 2019, adjusted 3-year overall survival was highest in the VHA (50%), followed by Academic/Research programs (46%), with lower survival in Integrated Network (40%), Comprehensive Community (39%), and Community programs (34%).

Stage-stratified analyses incorporating NCDB facility designation showed comparable stage-specific survival between VHA and NCDB Academic/Research Programs across all stages by 2019, with both outperforming all other NCDB facility types (Figure S2).

In a direct comparison between VHA and Academic/Research programs with model exclusion of other NCDB facility types (Figure S3), Academic/Research centers demonstrated slightly higher survival early in the study period (28% vs 24% in the VHA); however, survival gains were steeper within, and by 2019 VHA survival (50%) exceeded that of NCDB-Academic/Research programs (46%).

## Discussion

In this large, retrospective cohort study comparing patients with NSCLC in the VHA and the general US population, adjusted 3-year overall survival improved significantly in both cohorts between 2007 and 2019. However, gains were more pronounced in the VHA, culminating in a 10-percentage-point absolute survival advantage by 2019. This survival advantage began to emerge around 2012 and widened over time.

Improved stage distribution likely contributed to the overall survival gains observed in the VHA, consistent with prior findings from both our group and others. Specifically, our earlier work demonstrated that veterans were increasingly diagnosed at earlier stages of NSCLC over time,^17^ a trend also described in a national VHA study by Moghanaki et al.^16^ and others.^15^ However, stage-stratified analyses in the present study demonstrated greater improvements in 3-year overall survival over time among veterans, suggesting that earlier diagnosis alone does not fully explain the observed differences.

In sensitivity analyses, findings were consistent across both insurance type- and facility-based comparisons. When restricting the NCDB cohort to the most comprehensive insurance types (Medicare or private insurance), the VHA survival advantage persisted, with greater improvements in adjusted 3-year overall survival over time. Similarly, in analyses incorporating NCDB facility designations, both VHA and NCDB-Academic/Research programs demonstrated higher adjusted overall survival than less integrated NCDB facility types. By 2019, stage-specific survival was comparable between VHA and Academic/Research programs across all stages, perhaps indicating that integrated care delivery systems are associated with improved outcomes relative to more fragmented settings. However, overall survival gains were steeper within the VHA, and VHA survival exceeded that of NCDB-Academic/Research programs in later years. Together, these findings suggest that systems features beyond care integration alone, including coordinated wraparound services and sustained publicly funded infrastructure, may contribute to the greater population-level survival gains observed within the VHA.

Several system-level features unique to the VHA may explain these differences. As the largest integrated health-care system in the United States, the VHA benefits from centralized oversight and coordinated national leadership, including programs such as the National Oncology Program (NOP),^24^ which supports quality improvement, data monitoring, and care standardization across sites.^25^ The VHA also maintains comprehensive population-based registries through the Corporate Data Warehouse^26^ and VA Central Cancer Registry^27^ while operating a unified electronic health record that facilitates longitudinal tracking of care.^28^ Clinical pathways are developed nationally with expert provider input and disseminated uniformly to guide evidence-based practice.^29^ Veterans receive care in facilities that support coordinated interprofessional services, including universal access to tumor boards, thoracic oncology clinics, and care navigation.^30-32^ Many VHA sites also maintain formal academic affiliations, which support access to clinical trials and knowledge transfer between institutions.^33^ These features align with definitions of high-quality health systems—those that consistently deliver competent care, supported by effective monitoring, feedback, and patient-centered processes.^34^ Indeed, compared with the US general population, the VHA care delivery infrastructure has been associated with equal or superior performance across a range of health-care conditions.^13^^,^^35-38^

These high-level system structures translate to tangible benefits at the patient level. Unlike many civilian systems, the VHA provides comprehensive services with very low patient cost-sharing obligations, reducing financial barriers to timely lung cancer screening, diagnosis, treatment, and follow-up. This observation is consistent with prior work demonstrating that patients receiving care within universally accessible systems, such as the US Military Health System, experience improved lung cancer survival compared with the general population across all insurance types, including those with private coverage.^39^

Furthermore, the VHA provides key wraparound services, including transportation assistance, care navigation, psychosocial support, and coordinated survivorship care, which help structural barriers to care and support timely access to both curative and palliative treatment pathways.^15^^,^^36^^,^^40^^,^^41^ These programs are not peripheral but, rather, integral to the VHA’s delivery of high-quality cancer care,^40^ particularly for a high-risk population with significant medical and social complexity.^42^ Furthermore, the VHA has expanded its efforts to screen unmet social needs and connect patients to resources through system-wide investments in staffing and infrastructure.^43^^,^^44^

These findings carry implications for US health-care policy. The VHA’s integrated infrastructure, data systems, and wraparound services offer a viable model for delivery high-quality equitable cancer care at scale. Presently, the Lung Precision Oncology Program (LPOP) represents the cornerstone of the VHA’s lung cancer strategy. The LPOP connects veterans to centralized expertise in screening, molecular testing, and clinical trials through a hub-and-spoke design.^45^^,^^46^ The LPOP is supported by permanent institutional infrastructure rather than short-term funding mechanisms. National investment in staffing and data systems has enabled the consistent implementation of evidence-based practices, real-time data collection, and system-wide quality improvement across all sites. However, these systems remain vulnerable to shifts in federal priorities. Although the VHA is partially shielded from market-based pressures, its funding remains subject to congressional budgetary appropriations.^47^ Reductions in support for wraparound services or infrastructure could jeopardize the very systems that have enabled gains in both diagnosis and survival in this study and other recent VHA analyses.^16^^,^^17^ Sustained investment in these services is critical to preserving these functions and ensuring continued progress.

Finally, it is worth noting that the widening survival differences observed in the later years of the study period coincide temporally with the COVID-19 pandemic. Prior US-based studies documented substantial disruptions in cancer diagnosis and oncology care delivery during this period,^48-50^ while the Veterans Health Administration reported efforts to maintain continuity of cancer care through operational adaptations.^51-53^ This study has several strengths, including the use of large nationally representative cohorts and long-term survival analysis spanning over a decade of patient data. However, there are several limitations. Although we adjust for several clinically relevant factors, there may be unmeasured residual confounders. Additionally, although the NCDB is widely used to assess population-level cancer outcomes, it is limited to Commission on Cancer–accredited facilities and may not fully represent care received in under-resourced facilities.

In conclusion, this study demonstrates that NSCLC survival has improved nationally, with more substantial gains observed in the VHA compared with the general population. These findings underscore the importance of integrated care models and reinforce the role of system-wide infrastructure in achieving better cancer outcomes. The VHA offers a model of sustained investment with a measurable public health impact, underscoring the need for policies that preserve and expand the system features that make such outcomes possible.

## Supplementary Material

pkag054_Supplementary_Data

## Data Availability

The data underlying this study are not publicly available due to restrictions on access to protected health information. Veterans Health Administration (VHA) data were obtained from the VA Corporate Data Warehouse through the VA Informatics and Computing Infrastructure (VINCI) under an approved research protocol and are accessible only to investigators with appropriate VA appointments and approvals. National Cancer Database (NCDB) data were obtained through a data use agreement with the American College of Surgeons and the American Cancer Society and are available to qualified researchers upon application to the NCDB, subject to their data access policies. Analytic code may be made available from the corresponding author upon reasonable request, contingent on institutional approvals.
