# Supplementary material for: Trends in lung cancer survival: superior outcomes in US Veterans compared with the general population
Source: JNCI Cancer Spectr. 2026 May 21;10(4):pkag054. doi: 10.1093/jncics/pkag054 (PMC13426908; doi:10.1093/jncics/pkag054)

Supplementary Material

Table S1 - Patient characteristics of the overall unmatched study cohorts based on facility type designation						
**Variable**	**VA (N=79,027)**	**NCDB - Community cancer center (N=106,207)**	**NCDB - Comprehensive Community cancer center (N=564,039)**	**NCDB – Academic/Research cancer center (N=429,374)**	**NCDB - Integrated cancer center (N=278,715)**	**p-value**
Age in years, mean (SD)	66.69 (12.76%)	69.12 (10.38%)	69.52 (10.21%)	67.96 (10.35%)	69.43 (10.29%)	<0.0001
Race, n (%)						<0.0001
Non-White	16,992 (19.84%)	12,415 (11.69%)	66,203 (11.74%)	88,983 (20.72%)	41,731 (14.97%)	
White	68,669 (80.16%)	93,792 (88.31%)	497,836 (88.26%)	340,391 (79.28%)	236,984 (85.03%)	
Sex						<0.0001
Female	5,433 (6.34%)	48,372 (45.55%)	266,154 (47.19%)	207,212 (48.26%)	135,113 (48.48%)	
Male	80,216 (93.66%)	57,835 (54.45%)	297,885 (52.81%)	222,162 (51.74%)	143,602 (51.52%)	
Charlson-Deyo Comorbidity score, n (%)						<0.0001
0	31,612 (36.90%)	60,031 (56.52%)	310,266 (55.01%)	263,827 (61.44%)	153,886 (55.21%)	
1	21,358 (24.93%)	28,753 (27.07%)	157,842 (27.98%)	104,793 (24.41%)	76,574 (27.47%)	
2	13,650 (15.93)	11,426 (10.76%)	62,225 (11.03%)	39,098 (9.11%)	30,727 (11.02%)	
3+	19,041 (22.23%)	5,997 (5.65%)	33,706 (5.98%)	21,656 (5.04%)	17,528 (6.29%)	
Year of diagnosis, mean (SD)	2012.55 (3.55%)	2013.18 (3.71%)	2013.20 (3.70%)	2013.37 (3.66%)	2013.14 (3.69%)	<0.0001
Stage of cancer, n (%)						<0.0001
I	24,293 (28.36%)	21,555 (20.30%)	154,950 (27.47%)	131,331 (30.59%)	77,855 (27.93%)	
II	7,680 (8.97%)	9,388 (8.84)	52,286 (9.27%)	38,807 (9.04%)	24,791 (8.89%)	
III	19,361 (22.60%)	25,823 (24.31%)	122,857 (21.78%)	87,381 (20.35%)	59,885 (21.49%)	
IV	34,327 (40.07%)	49,441 (46.55%)	233,946 (41.48%)	171,885 (40.02%)	116,184 (41.69%)	

Figure S1. Adjusted 3-year overall survival among patients with NSCLC by facility type, including Veterans Health Administration (VHA) and standard National Cancer Database (NCDB) facility categories (Academic/Research Programs, Integrated Network Cancer Programs, Comprehensive Community Cancer Programs, and Community Cancer Programs), 2007–2019. Estimates are reported with 95% confidence intervals. P-value corresponds to the interaction between facility type and year of diagnosis (spline × facility), reflecting differences in survival trends over time.

Figure S2. Adjusted 3-year overall survival by stage at diagnosis (Panel A - stage I; Panel B - stage II; Panel C - stage III; Panel D - stage IV) among patients with NSCLC by facility type, with comparison based on facility type, 2007–2019. Estimates are reported with 95% confidence intervals. P-value corresponds to the interaction between facility type and year of diagnosis (spline × facility), reflecting differences in survival trends over time.

Figure S3. Adjusted 3-year overall survival among patients with NSCLC comparing VHA and NCDB-Academic/Research Programs with exclusion of other NCDB facility types, 2007–2019. Estimates are reported with 95% confidence intervals. P-value corresponds to the interaction between facility type and year of diagnosis (spline × facility), reflecting differences in survival trends over time.

Supplementary Material - Figure S1

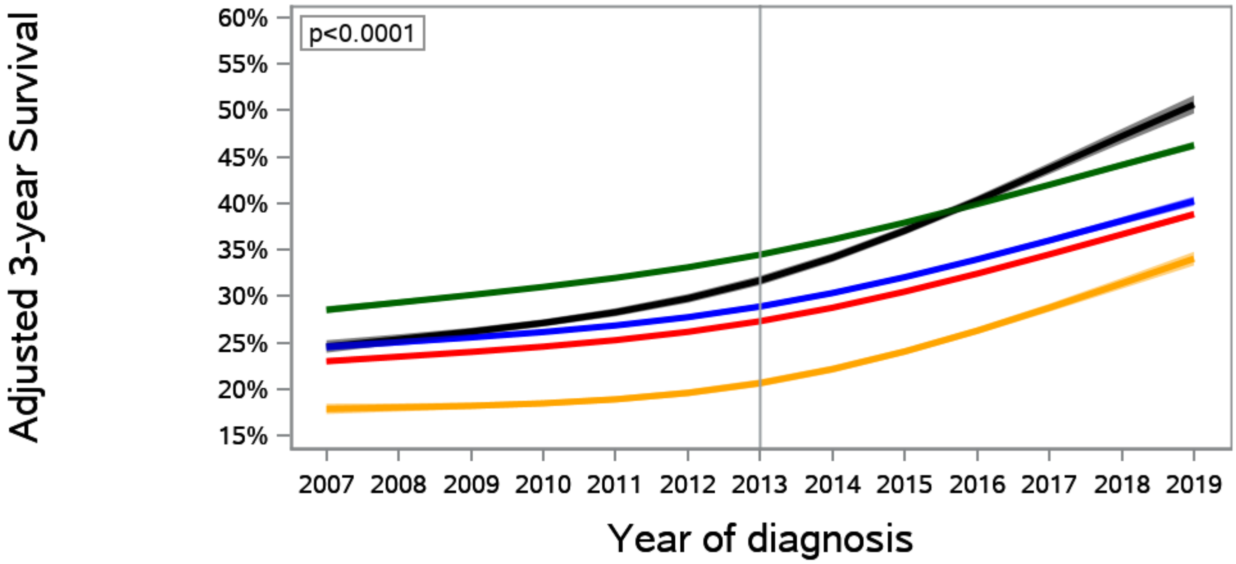

	2007	2008	2009	2010	2011	2012	2013	2014	2015	2016	2017	2018	2019
VHA	25%	25%	26%	27%	28%	30%	32%	34%	37%	40%	44%	47%	51%
NCDB- Academic/Research	29%	29%	30%	31%	32%	33%	34%	36%	38%	40%	42%	44%	46%
NCDB- Integrated Network	25%	25%	26%	26%	27%	28%	29%	30%	32%	34%	36%	38%	40%
NCDB- Comprehensive Community	23%	24%	24%	25%	25%	26%	27%	29%	31%	32%	35%	37%	39%
NCDB- Community	18%	18%	18%	18%	19%	20%	21%	22%	24%	26%	29%	31%	34%

Supplementary Material - Figure S2A

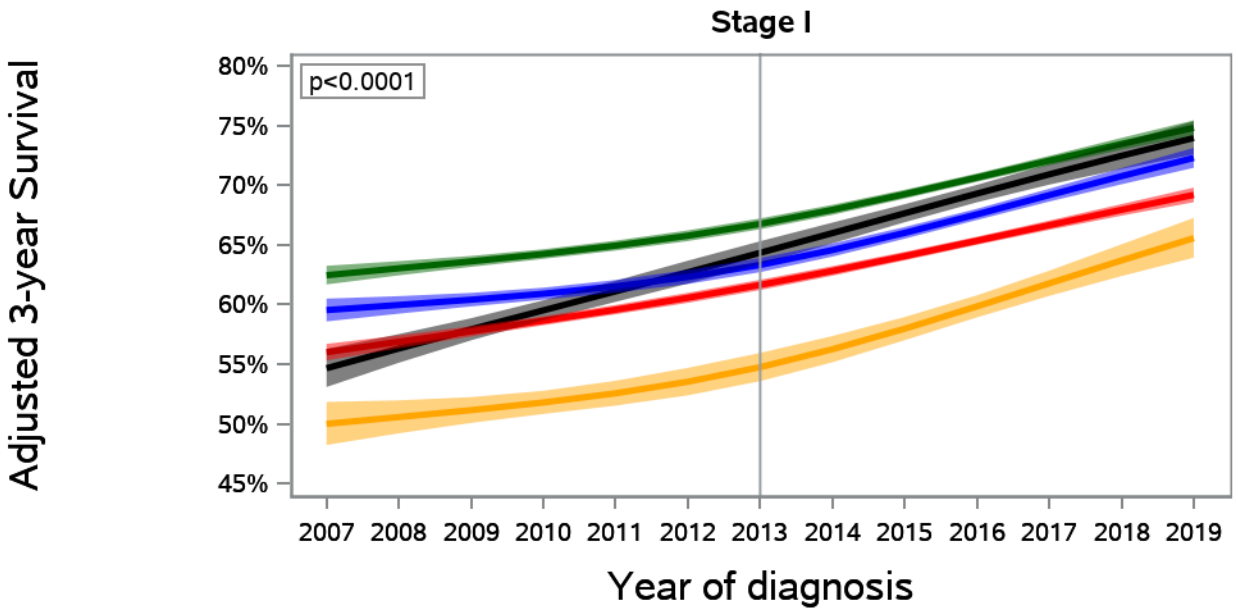

	2007	2008	2009	2010	2011	2012	2013	2014	2015	2016	2017	2018	2019
VHA	**55%**	**56%**	**58%**	**60%**	**61%**	**63%**	**64%**	**66%**	**68%**	**69%**	**71%**	**72%**	**74%**
NCDB- Academic/Research	**62%**	**63%**	**64%**	**64%**	**65%**	**66%**	**67%**	**68%**	**69%**	**71%**	**72%**	**73%**	**75%**
NCDB- Integrated Network	**60%**	**60%**	**60%**	**61%**	**62%**	**62%**	**63%**	**65%**	**66%**	**68%**	**69%**	**71%**	**72%**
NCDB- Comprehensive Community	**56%**	**57%**	**58%**	**59%**	**60%**	**61%**	**62%**	**63%**	**64%**	**65%**	**67%**	**68%**	**69%**
NCDB- Community	**50%**	**51%**	**51%**	**52%**	**53%**	**54%**	**55%**	**56%**	**58%**	**60%**	**62%**	**64%**	**66%**

Supplementary Material - Figure S2B

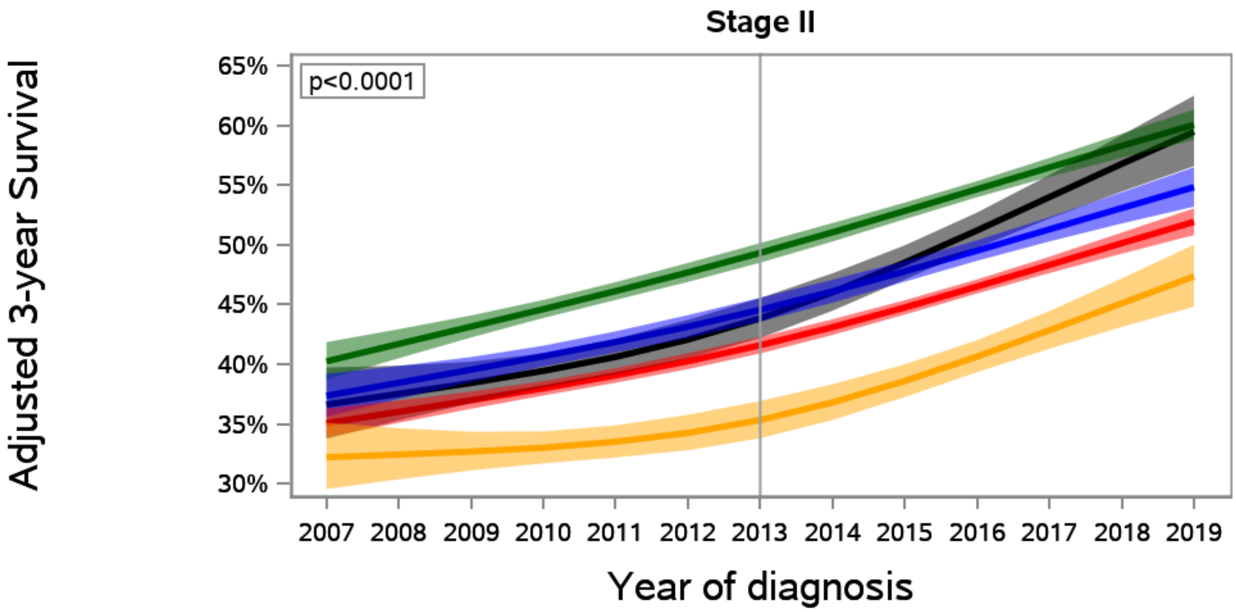

	2007	2008	2009	2010	2011	2012	2013	2014	2015	2016	2017	2018	2019
VHA	37%	38%	38%	39%	41%	42%	44%	46%	49%	51%	54%	57%	59%
NCDB- Academic/Research	40%	42%	43%	45%	46%	48%	49%	51%	53%	55%	56%	58%	60%
NCDB- Integrated Network	37%	38%	40%	41%	42%	43%	45%	46%	48%	50%	51%	53%	55%
NCDB- Comprehensive Community	35%	36%	37%	38%	39%	40%	42%	43%	45%	47%	48%	50%	52%
NCDB- Community	32%	32%	33%	33%	34%	34%	35%	37%	39%	41%	43%	45%	47%

Supplementary Material - Figure S2C

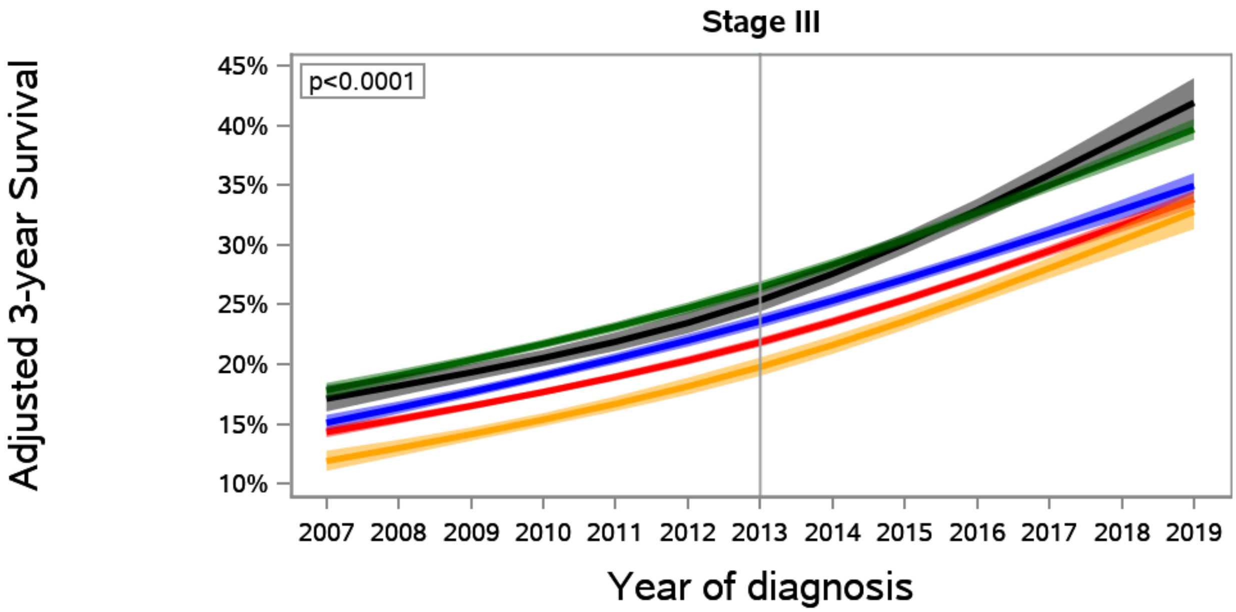

	2007	2008	2009	2010	2011	2012	2013	2014	2015	2016	2017	2018	2019
VHA	17%	18%	19%	21%	22%	23%	25%	28%	30%	33%	36%	39%	42%
NCDB- Academic/Research	18%	19%	20%	22%	23%	25%	26%	28%	30%	33%	35%	37%	40%
NCDB- Integrated Network	15%	16%	18%	19%	20%	22%	24%	25%	27%	29%	31%	33%	35%
NCDB- Comprehensive Community	14%	15%	17%	18%	19%	20%	22%	24%	25%	27%	29%	32%	34%
NCDB- Community	12%	13%	14%	15%	17%	18%	20%	22%	24%	26%	28%	30%	33%

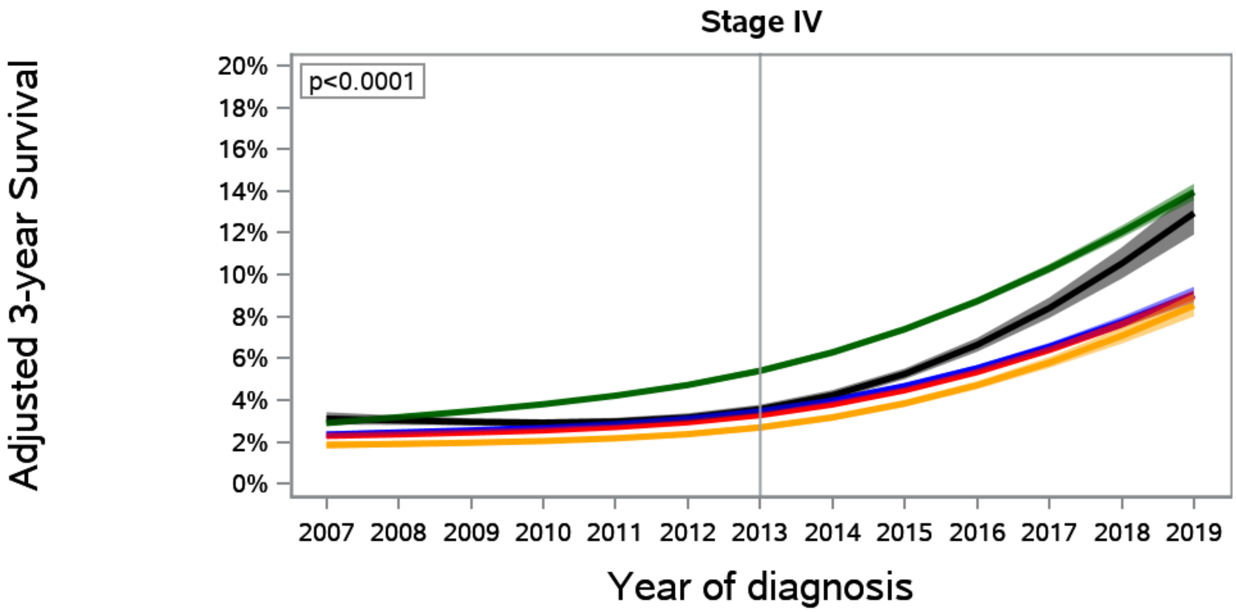

	2007	2008	2009	2010	2011	2012	2013	2014	2015	2016	2017	2018	2019
VHA	3%	3%	3%	3%	3%	3%	4%	4%	5%	7%	8%	11%	13%
NCDB- Academic/Research	3%	3%	3%	4%	4%	5%	5%	6%	7%	9%	10%	12%	14%
NCDB- Integrated Network	2%	2%	3%	3%	3%	3%	3%	4%	5%	6%	7%	8%	9%
NCDB- Comprehensive Community	2%	2%	2%	3%	3%	3%	3%	4%	4%	5%	6%	8%	9%
NCDB- Community	2%	2%	2%	2%	2%	2%	3%	3%	4%	5%	6%	7%	9%

Supplementary Material - Figure S3

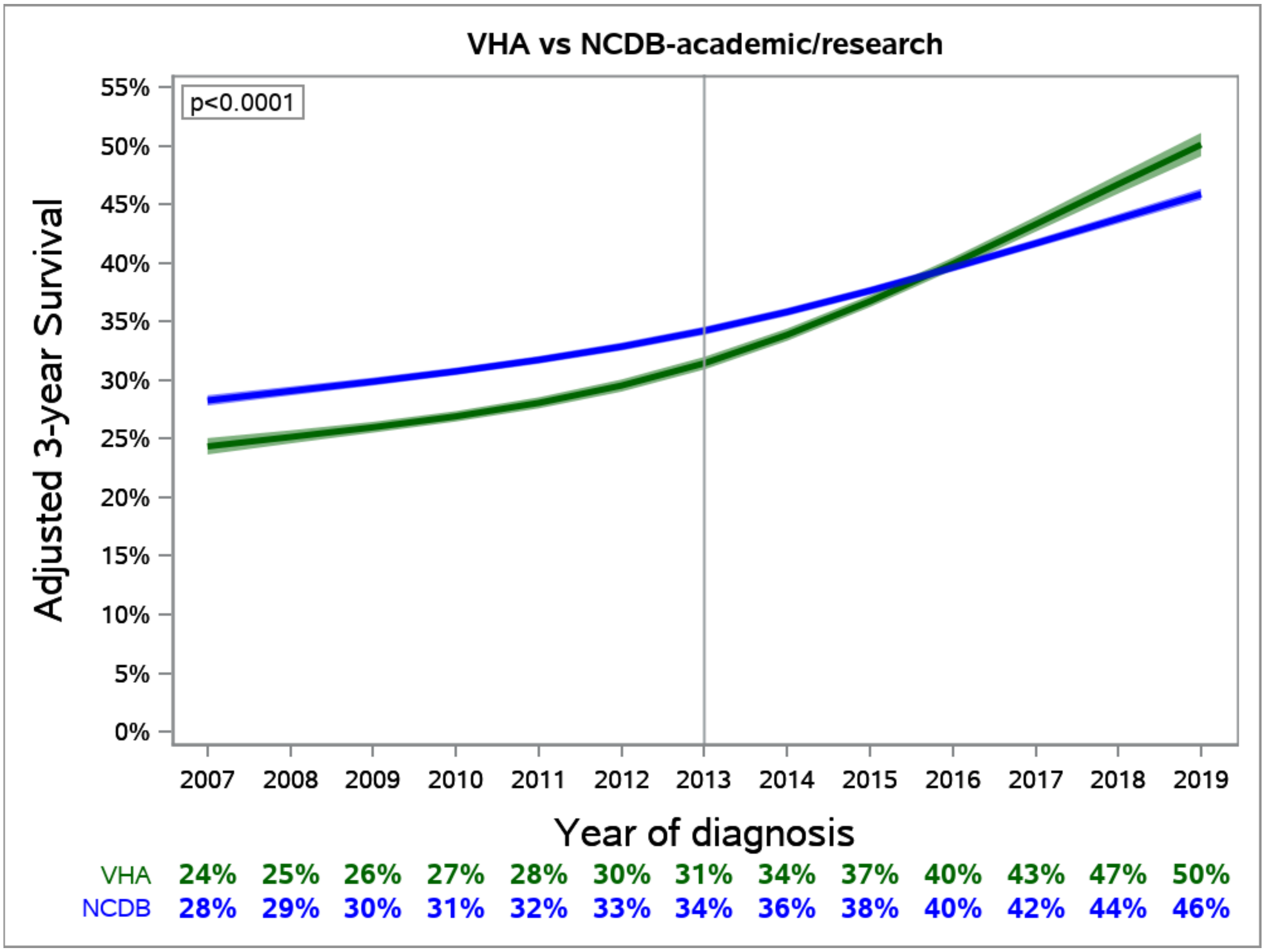

Supplement: pkag054_Supplementary_Data [file pkag054_supplementary_data.pdf]
